# 3D Visualization of Individual Regenerating Retinal Ganglion Cell Axons Reveals Surprisingly Complex Growth Paths

**DOI:** 10.1523/ENEURO.0093-17.2017

**Published:** 2017-08-29

**Authors:** Eric R. Bray, Markus Noga, Kinjal Thakor, Yunfang Wang, Vance P. Lemmon, Kevin K. Park, Pantelis Tsoulfas

**Affiliations:** Department of Neurological Surgery, Miami Project to Cure Paralysis, University of Miami Miller School of Medicine, Miami, FL 33136

**Keywords:** axon, clearing, CNS, iDISCO, regeneration, RGC

## Abstract

Retinal ganglion cells (RGCs), the sole output cells of the retina, are a heterogeneous population of neurons that project axons to visual targets in the brain. Like most CNS neurons, RGCs are considered incapable of mounting long distance axon regeneration. Using immunolabeling-enabled 3D imaging of solvent-cleared organs (iDISCO) in transgenic mice, we tracked the entire paths of individual RGC axons and show that adult RGCs are highly capable of spontaneous long-distance regeneration, even without any treatment. Our results show that the Thy1-H-YFP mouse sparsely labels RGCs, consisting predominantly of regeneration-competent α-type RGCs (αRGCs). Following optic nerve crush, many of the YFP-labeled RGC axons extend considerable distances proximal to the injury site with only a few penetrating through the lesion. This tortuous axon growth proximal to the lesion site is even more striking with intravitreal ciliary neurotrophic factor (CNTF) treatment. We further demonstrate that despite traveling more than 5 mm (i.e., a distance equal to the length of mouse optic nerve), many of these circuitous axons are confined to the injury area and fail to reach the brain. Our results re-evaluate the view that RGCs are naturally incapable of re-extending long axons, and shift the focus from promoting axon elongation, to understanding factors that prevent direct growth of axons through the lesion and the injured nerve.

## Significance Statement

Retinal ganglion cells (RGCs) are viewed as being incapable of mounting lengthy axon regeneration. Using whole tissue immunolabeling, we establish a technique to visualize and trace the entire paths of small populations of genetically labeled RGC axons as they regenerate. Following optic nerve injury, few axons grow beyond the lesion, but we find these axons branch and form loops proximal to the lesion. A regeneration inducing treatment further exacerbates branching and tortuous growth, while only modestly increasing the number of RGC axons that successfully grow beyond the lesion. Our study demonstrates extensive and circuitous RGC axon elongation both in pre- and post-lesion regions, highlighting the need to better understand the factors that inhibit direct axon growth in the optic nerve.

## Introduction

Lack of axon regeneration is a major obstacle preventing functional recovery after axon injury. Like other neurons in the CNS, it is thought that retinal ganglion cells (RGCs) have a limited ability to regenerate spontaneously. Nonetheless, growth factors or modification of genes promote RGC axonal regeneration, to some extent. For example, supplying RGCs with cytokines, or genetic modification of *Pten*, *Pcaf*, *Stat3*, *Socs3*, *c-Myc*, *dcxl*, or *Klf4* allows some RGC axons to regenerate with few axons reaching the brain targets (Park et al., 2008; Moore et al., 2009; Smith et al., 2009; Puttagunta et al., 2014; Belin et al., 2015; Nawabi et al., 2015; Leibinger et al., 2016; Mehta et al., 2016).

Multiple reports have demonstrated that some regenerating RGC axons travel circuitously within the optic nerve after intraorbital crush injury (Luo et al., 2013; Pernet et al., 2013). However, these studies have used anterograde tracers that label all RGC axons, making it difficult to identify individual fibers and visualize how axons of different RGC types behave as they regenerate. Here we sought to combine sparse neuronal labeling with the immunolabeling-enabled 3D imaging of solvent-cleared organs (iDISCO) technique (Renier et al., 2016) and trace the entire path of individual axons as they regenerate.

RGCs are a heterogeneous population of neurons. They are divided into several subclasses based on their morphologic, physiologic, and molecular properties. Previous studies have shown that different RGC types differ in their ability to regenerate axons. For instance, studies in cats have shown that αRGCs regenerate axons better than other RGC types (Watanabe and Fukuda, 2002). Similarly, in mice it was demonstrated that αRGCs have greater propensity to regenerate axons after crush injury (Duan et al., 2015). RGCs in the Thy1-H-YFP mouse line have a “Golgi-like” labeling, allowing for the characterization of their dendrites and axons. Importantly, this line labels few RGCs, most of which are immune-positive for SMI-32, a marker of αRGCs (Feng et al., 2000; Lin et al., 2004; Coombs et al., 2006).

In this study, we first validate that the Thy1-H-YFP mouse sparsely labels αRGCs. We used a combination of confocal imaging and iDISCO to analyze the dendrites and axons of the labeled neurons. We find that, after optic nerve crush, (1) αRGC dendrites decrease in complexity, and the dendritic arbors are even less complex in animals treated with an axon growth promoting-stimulator (i.e., CNTF); (2) innately, axons remodel and regrow extensively proximal to the crush site; and (3) following CNTF, axon elongation is extremely circuitous, and these axons never reach the brain, despite growing distances that would allow them to reach the brain, had they grown straight. Overall, our results show that RGCs can re-extend axons very well, even in the absence of a growth stimulator, but their inability to traverse the lesion area along with meandering axon growth limit “meaningful” regeneration, dramatically reducing the number of RGCs successfully regenerating axons into the brain. Our results shift the focus from promoting axon elongation, to understanding factors that prevent direct growth of CNS axons through the injured nerve.

## Materials and Methods

All experimental procedures were performed in compliance with protocols approved by the Institutional Animal Care and Use Committee at the University of Miami. The Thy1-H-YFP mouse strain was used for all experiments (The Jackson Laboratory stock number 003782). All animals were housed in a viral antigen-free facility and kept under standard 12/12 h light/dark cycle conditions. For all surgical procedures, mice where anaesthetized with ketamine and xylazine. For analgesia, buprenorphine (0.05 mg/kg) was administered postoperatively. The exact number of animals used for each group is in the main text and figure legends.

### Intravitreal injection

Female and male mice six to eight weeks old underwent unilateral intravitreal adeno-associated virus serotype 2 (AAV) injection. AAVs carried expression constructs for ciliary neurotrophic factor (CNTF; AAV-CNTF; Yungher et al., 2015) or placental alkaline phosphatase (PLAP) as a control transgene (AAV-PLAP). AAversus were made by the University of Miami's Viral Vector Core. Typical titers were 5 × 10^12^ GC/ml. A fine glass micropipette was inserted into the posterior chamber taking care to avoid damaging the lens. Using a Hamilton syringe (Hamilton 80900) 2 µl of virus was slowly injected. Cholera toxin β (CTB) conjugated to Alexa Fluor 594 (ThermoFisher C34777, 2 µg/µl in PBS) was injected as described above. CTB was injected 1 h postcrush in the 3 d postcrush (3dpc) group.

### Optic nerve crush

Animals received unilateral optic nerve crush. Time points postcrush included in this study: 3 dpc, 3 weeks postcrush (3wpc), and 6wpc. AAV was injected 7 d before crush in the 3dpc group, or 3 d before crush in the 3wpc and 6wpc groups. For the optic nerve crush procedure, the optic nerve was exposed intra-orbitally by blunt dissection. The optic nerve was crushed with forceps (#5 Dumont, Fine Science Tools) for 10 s ∼1 mm distal to the emergence from the globe.

### Immunohistochemistry

Mice were killed by transcardial perfusion with ice cold PBS and 4% paraformaldehyde. The optic nerve was cut proximal to the optic chiasm. The globe with attached optic nerve was postfixed in 4% paraformaldehyde overnight at 4°C. For retinal whole mount staining, the retina was carefully dissected out of the globe and derestricting cuts were placed in each quadrant. Tissue was washed with PBS and blocked in 5% normal donkey serum (Sigma D9663) in PBS + 0.3% Triton X-100 (PBST). Tissue was then incubated in blocking buffer containing primary antibodies, overnight at 4°C. Primary antibodies: goat-anti-osteopontin (OSPN) 1:500 (R&D Systems, AF808), rabbit-anti-melanopsin (OPN4; UF006) 1:2500 (Advanced Targeting Systems, AB-N38), goat-anti-GFP 1:1000 (Abcam, ab6673), rabbit-anti-GFP 1:1000 (Millipore, ab3080), and rabbit-anti-cocaine- and amphetamine-regulated transcript (CART; 55–102) 1:1000 (Phoenix Pharmaceuticals, H-003-62). Following incubation with primary antibody, tissue was washed extensively in PBST. Tissue was then incubated in blocking buffer containing secondary antibodies 1:500, overnight at 4°C. Secondary antibodies: donkey-anti-rabbit Alexa Fluor 488 (ThermoFisher, A-21206), donkey-anti-rabbit Alexa Fluor 647 (ThermoFisher, A-31573), donkey-anti-goat Alexa Fluor 488 (ThermoFisher, A-11055), and donkey-anti-goat Alexa Fluor 647 (ThermoFisher, A-21447). Following secondary incubation retinas were extensive washed with PBST, mounted with Slowfade (ThermoFisher, S36973) and coverslipped. Imaging was performed on an Olympus FV1000 confocal microscope, objectives: UPlanSApo 10 × 0.40 N.A. and UPlanFLN 40 × 1.3 N.A., and Olympus FV10-ASW Ver 0.200C software. Images were analyzed using Imaris software (Bitplane). Figures were composed using Photoshop CS6 (Adobe) and Illustrator CS5 (Adobe).

### RGC quantification

The number of YFP^+^ RGCs per retina was quantified from low-magnification z-stack images that tiled the entire retina using an Olympus Fluoview 1000 confocal microscope. RGCs were defined by: soma location in the ganglion cell layer or inner nuclear layer (displaced RGC), and the presence of an axon. To determine the subtype of YFP^+^ RGCs, every YFP^+^ RGC in a retina was assessed for immunoreactivity with a RGC subtype marker: OSPN, Opn4, or CART (see Results for *n* number of retinas assessed for each marker). Survival of YFP^+^ RGCs was determined as the number of YFP^+^ RGCs remaining or the percentage of YFP^+^ RGCs remaining compared to the uninjured contralateral retina. To analyze RGC dendrites, high-magnification z-stack images were taken of individual RGCs from the retinal nerve fiber layer to the inner nuclear layer. Images were tiled and reconstructed if a RGC’s dendrites extended out of field. Dendrites were traced using ImageJ/FIJI and the Simple Neurite Tracer plugin (Longair et al., 2011; Schindelin et al., 2012). Linear Sholl analysis was completed using the Sholl analysis plugin for ImageJ/FIJI (Ferreira et al., 2014). Sholl analysis data were analyzed as the average number of intersections observed in radii bins of 30 µm. Data were preprocessed using R 3.3.1 (https://cran.r-project.org/).

### iDISCO

For whole mount staining and clearing we use the enhanced version of iDISCO. For a full description of the protocol see, Renier et al. (2014), Renier et al. (2016), and website (http://lab.rockefeller.edu/tessier-lavigne/assets/file/whole-mount-staining-bench-protocol-january-2015.pdf). Dissected optic nerves were dehydrated with a methanol/PBS series, 20%, 40%, 60%, 80%, and 100%, bleached overnight with 5% H_2_O_2_ in 100% methanol at 4°C. Rehydrated with a methanol series in PBS and 0.2% Triton X-100, 80%, 60%, 40%, 20%, and 0%. Incubated with 1× PBS, 0.2% Triton X-100, 20% DMSO, and 0.3 M glycine, 37°C for 2 d. Block in 1× PBS, 0.2% Triton X-100, 10% DMSO, and 6% donkey serum, 37°C, for 2 d. Wash in 1× PBS, 0.2% Tween 20 with 10 μg/ml heparin (PTwH), room temperature for 1 h, twice. Incubate with a chicken IgY recognizing GFP epitopes (Aves, #GFP-1020, 1:200) in PTwH, 5% DMSO, and 3% donkey serum, 37°C, for 2 d. Wash in PTwH for 10 min, 15 min, 30 min, and 1 h then 2 h or longer to the next day. Incubate with a goat-anti-chicken Alexa Fluor 488 1:300 (ThermoFisher, A-11039) in PTwH and 3% donkey serum, 37°, 2 d. Wash in PTwH for 10, 15, 30, and 60 min each and then 2 h or longer for 2 d. After the final wash, the samples were cleared.

### Clearing

Washed samples were incubated at room temperature with shaking. First, for 1 h for each step with 20%, 40%, 60%, and 80% methanol in water followed by 30 min in 100% methanol twice. Next, they were incubated for 3 h in 66% dichloromethane (DCM) and 33% methanol then 20 min in 100% DCM, twice. Final clearing solution was in dibenzylether (DBE) with no shaking. Cleared optic nerves were mounted onto a cover glass with DBE and imaged on an Olympus confocal microscope (Fluoview 1000) using a 20× UPlanSApo objective (N.A. = 0.75). We used an optical zoom of 1.4× and each optic section was 1–1.2 μm. Individual stacks of images were stitched using the program XuvStitch 1.8.099x64 (http://www.xuvtools.org/doku.php).

### Axon analysis

Reconstructed confocal images were analyzed using the FilamentTracer function in Imaris 8.4.1 (Bitplane). To be included in quantitative analysis an axon had to be traced from the proximal optic nerve head to its termination. An axon also had to be resolvable from surrounding axons. FilamentTracer statistics were exported and preprocessed using R 3.3.1 (https://cran.r-project.org/).

### Statistical analysis

Data preprocessing was conducted using R 3.3.1 (https://cran.r-project.org/). Statistical analysis and graph creation was performed with Prism 6 (GraphPad Software). See Table 1 for the list of statistical tests used.

**Table 1.** Summary of statistics

	Data structure	Type of test	Observed power (α = 0.05)
Results text	Dependent continuous	Pearson correlation	0.0479
Figure 2*K*	3 groups, normal distribution	ANOVA	
Uninjured vs 6wpc	Normal distribution	*Post hoc*: Tukey's multiple comparisons	0.7877
Uninjured vs CNTF + 6wpc	Normal distribution	*Post hoc*: Tukey's multiple comparisons	0.0032
6wpc vs CNTF + 6wpc	Normal distribution	*Post hoc*: Tukey's multiple comparisons	0.0214
Figure 2*L*	3 groups, normal distribution	ANOVA	0.5523
Figure 2*M*	3 groups, repeated measures, normal distribution	Two-way ANOVA	
Uninjured vs 6wpc	Normal distribution	*Post hoc*: Tukey's multiple comparisons	0.0188
Uninjured vs CNTF + 6wpc	Normal distribution	*Post hoc*: Tukey's multiple comparisons	<0.0001
6wpc vs CNTF + 6wpc	Normal distribution	*Post hoc*: Tukey's multiple comparisons	0.0353
Figure 2*M* (ANOVA at each distance)			
Distance: 30 μm	3 groups, normal distribution	ANOVA	
Uninjured vs 6wpc	Normal distribution	*Post hoc*: Tukey's multiple comparisons	0.0055
Uninjured vs CNTF + 6wpc	Normal distribution	*Post hoc*: Tukey's multiple comparisons	<0.0001
6wpc vs CNTF + 6wpc	Normal distribution	*Post hoc*: Tukey's multiple comparisons	0.0024
Distance: 60 μm	3 groups, normal distribution	ANOVA	
Uninjured vs 6wpc	Normal distribution	*Post hoc*: Tukey's multiple comparisons	0.0001
Uninjured vs CNTF + 6wpc	Normal distribution	*Post hoc*: Tukey's multiple comparisons	<0.0001
6wpc vs CNTF + 6wpc	Normal distribution	*Post hoc*: Tukey's multiple comparisons	0.0019
Distance: 90 μm	3 groups, normal distribution	ANOVA	
Uninjured vs 6wpc	Normal distribution	*Post hoc*: Tukey's multiple comparisons	0.0002
Uninjured vs CNTF + 6wpc	Normal distribution	*Post hoc*: Tukey's multiple comparisons	<0.0001
6wpc vs CNTF + 6wpc	Normal distribution	*Post hoc*: Tukey's multiple comparisons	0.0455
Distance: 120 μm	3 groups, normal distribution	ANOVA	
Uninjured vs 6wpc	Normal distribution	*Post hoc*: Tukey's multiple comparisons	0.1008
Uninjured vs CNTF + 6wpc	Normal distribution	*Post hoc*: Tukey's multiple comparisons	0.0014
6wpc vs CNTF + 6wpc	Normal distribution	*Post hoc*: Tukey's multiple comparisons	0.2399
Distance: 150 μm	3 groups, normal distribution	ANOVA	
Uninjured vs 6wpc	Normal distribution	*Post hoc*: Tukey's multiple comparisons	0.6984
Uninjured vs CNTF + 6wpc	Normal distribution	*Post hoc*: Tukey's multiple comparisons	0.1204
6wpc vs CNTF + 6wpc	Normal distribution	*Post hoc*: Tukey's multiple comparisons	0.4675
Figure 3*H*	Dependent continuous	Pearson correlation	<0.0001
Results text	2 groups, 2 outcomes	Fisher's exact text	0.0135
Figure 5*B* (6wpc vs CNTF + 6wpc)	Non-normal distribution	Mann Whitney test	0.0001
Figure 5*C*	Normal distribution, unequal variance	Welch's test	<0.0001
Figure 5*D*	Normal distribution, unequal variance	Welch's test	0.1152
Figure 5*E*	Normal distribution, unequal variance	Welch's test	<0.0001

## Results

### Thy1-H mouse sparsely labels subpopulation of αRGCs

**Figure 1.**
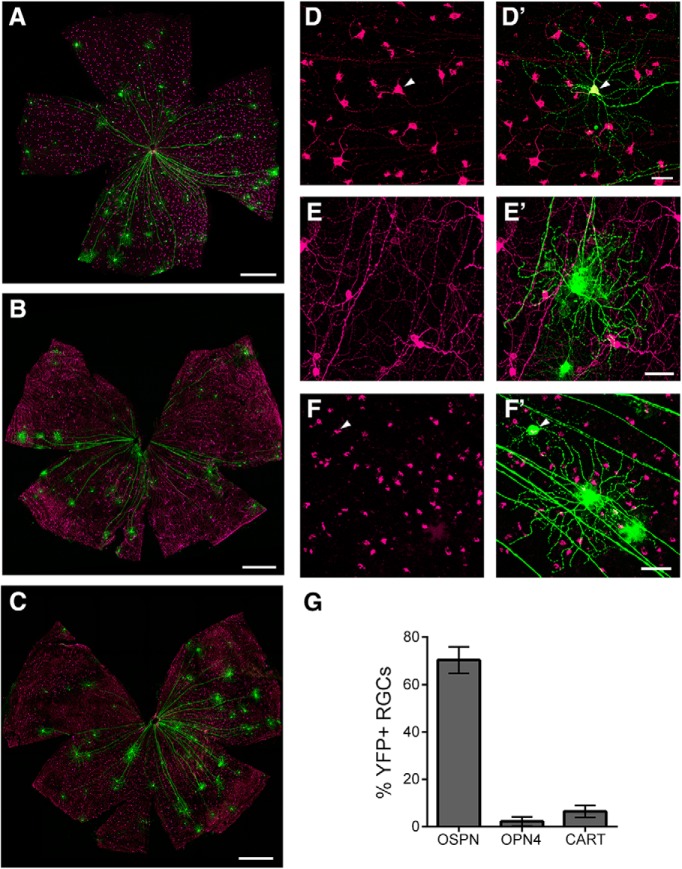
Identification of RGC subtypes labeled by the Thy1-H-YFP mouse line. Retinal whole mount preparations were immunostained for YFP (green) and markers of three RGC subtypes (magenta): OSPN (***A***, ***D***, ***D’***), OPN4 (***B***, ***E***, ***E’***), and CART (***C***, ***F***, ***F’***). ***A–C***, Low-magnification images of flat mount retina specimens that demonstrate the number of RGCs labeled by the Thy1-H-YFP mouse line as well as by each RGC subtype marker. Scale bar, 500 µm. ***D–F***, High-magnification images of RGCs labeled by each marker. ***D’–F’***, YFP^+^ RGCs (green) shown in the presence of different RGC subtypes (magenta). Arrowheads mark YFP^+^ RGCs immunoreactive for subtype marker. Scale bar, 50 µm. ***G***, The percentage of YFP^+^ RGCs that are immunoreactive for each marker. Bar graphs of mean ± SEM (retinas: OSPN *n* = 9, OPN4 *n* = 3, and CART *n* = 3).

To visualize and track the growth of individual axons in the unsectioned mouse optic nerve we used the Thy1-H-YFP transgenic mouse line, which sparsely labels RGCs, including the regeneration competent αRGCs (Feng et al., 2000; Coombs et al., 2006; Duan et al., 2015). We observed that there are ∼70 YFP^+^ RGCs in each flat-mounted retina of an adult Thy1-H-YFP mouse (*n* = 9 retina). While the number of YFP^+^ RGCs per retina varies between animals (σ = 21), there is a strong correlation (*R*^2^ = 0.9943, *p* < 0.05, *n* = 3 pairs of retina) between the left and right retina of an animal. To determine what portion of these YFP^+^ RGCs are αRGCs, we immunostained the retinas with an antibody against OSPN, a molecular marker of αRGCs (Duan et al., 2015; Sanes and Masland, 2015). We found that ∼70% of YFP^+^ RGCs are immunoreactive for OSPN (Fig. 1). To further define the molecular identity of YFP^+^ RGCs, we stained the retinas with antibodies against OPN4 (a marker of intrinsically photosensitive RGCs) and CART (a marker of direction selective RGCs). Very few YFP^+^ RGCs were immunoreactive for OPN4 (i.e., <2% of total YFP^+^ RGCs) or CART (i.e., <10% of total YFP^+^ RGCs; Fig. 1*A–G*). Taken together, these results demonstrate that the Thy1-H-YFP mouse line sparsely labels RGCs, which are primarily OSPN^+^.

**Figure 2.**
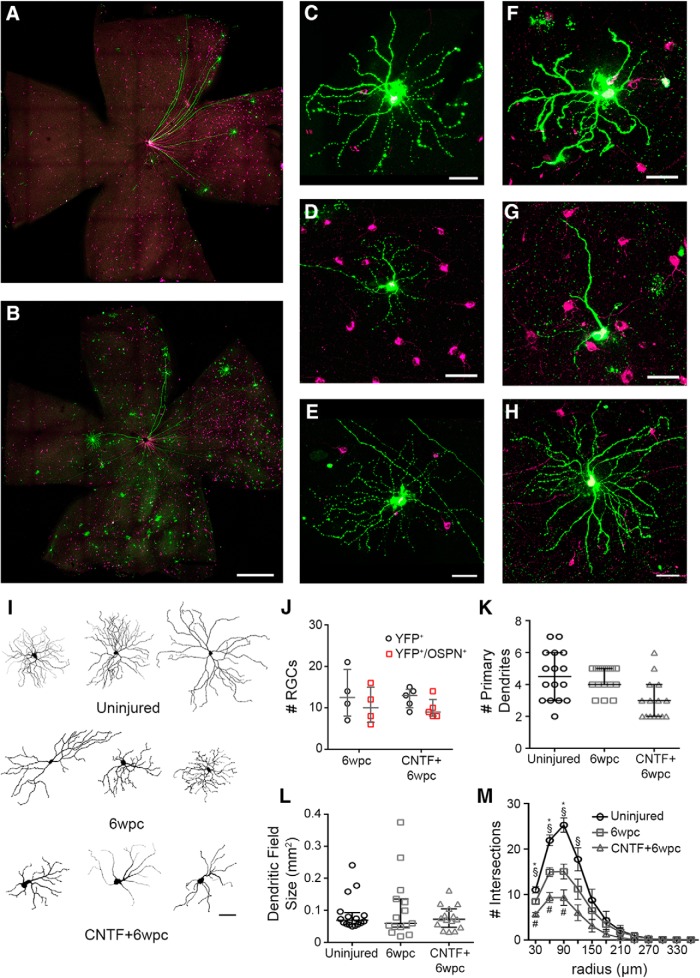
Response of YFP^+^ RGCs to axonal injury and AAV-CNTF injection. Six weeks following optic nerve crush (6wpc), retinal whole mounts from AAV-PLAP (6wpc; ***A***, ***C–E***)- and AAV-CNTF (CNTF + 6wpc)-injected (***B***, ***F–H***) animals were immunostained for YFP (green) and OSPN (magenta). ***A***, ***B***, Low-magnification images of retinal whole mount preparations show few YFP^+^ RGCs surviving six weeks following crush injury. Scale bar, 500 µm. RGCs were defined by: soma location in the ganglion cell layer or inner nuclear layer (displaced RGC), and the presence of an axon. ***C–H***, High-magnification images of YFP^+^/OSPN^+^ RGCs from 6wpc (***C–E***) and CNTF + 6wpc (***F–H***) animals. Three example RGCs are shown for each animal group. Scale bar, 50 µm. ***I***, Example traces of YFP^+^/OSPN^+^ RGC dendrites from each condition. Scale bar, 50 µm. ***J***, Quantification of YFP^+^ RGCs and YFP^+^/OSPN^+^ RGCs in each condition, each dot represents 1 retina (6wpc *n = 4*, CNTF + 6wpc *n = 5*). ***K***, Number of primary dendrites observed for each RGC (*p* < 0.05 6wpc vs CNTF + 6wpc, ANOVA with Tukey’s *post hoc*). ***L***, Dendritic field size for each RGC (mm^2^). ***J–L***, Bars, median and interquartile range. ***M***, Sholl analysis of RGC dendrites; bars, mean ± SEM (*p < 0.05 uninjured versus 6wpc, §*p* < 0.05 uninjured versus CNTF + 6wpc, #*p* < 0.05 6wpc versus CNTF + 6wpc, ANOVA with Tukey’s *post hoc* at each distance). ***K–M***, Uninjured *n* = 16 RGCs from five animals, 6wpc *n* = 15 RGCs from four animals, CNTF + 6wpc *n* = 15 RGCs from five animals).

To examine the fate of YFP^+^ RGCs in response to axon injury, we performed intraorbital optic nerve crush and evaluated their survival in the presence or absence of a growth promoting factor (Fig. 2*A–H*,*J*). Six weeks postcrush, ∼13 YFP^+^ RGCs survived (Fig. 2*J*, 6wpc). Of these, ∼10 were OSPN^+^ RGCs, which represents ∼76% of total remaining YFP^+^ RGCs (Fig. 2*J*). Several studies have shown that virally transferred CNTF promotes RGC survival and axon regeneration. AAV2 expressing a secreted form of CNTF did not alter the survival of YFP^+^ and YFP^+^/OSPN^+^ RGCs (Fig. 2*J*, CNTF + 6wpc). These data show that the majority of surviving YFP^+^ RGCs in the Thy1-H-YFP mouse are αRGCs. These findings underscore the utility of the Thy1-H-YFP mouse line for studying axon regeneration in a small number of regeneration competent RGCs, and establishes the feasibility of examining how a specific RGC type will behave after injury and CNTF.

### Changes in αRGC dendrite morphology following axon injury

In addition to investigating the survival of YFP^+^ RGCs, we examined the changes in dendrite morphology following intraorbitral optic nerve crush. Again, we focused on YFP^+^ αRGCs (i.e., YFP^+^/OSPN^+^ RGCs). Representative images of YFP^+^/OSPN^+^ RGCs and their dendrites in uninjured and injured (6wpc) animals are shown in Figures 1*D’–F’*, 2*C–E*,*I*, respectively. At six weeks following injury, we did not observe a significant change in dendritic field area or the number of primary dendrites compared to uninjured animals (Fig. 2*K*,*L*). Sholl analysis, which is commonly used to evaluate dendritic field arrangement and density (Sholl, 1953), shows that there is a significant reduction in dendrite complexity following intraorbital crush (Fig. 2*M*, uninjured versus 6wpc, *p < 0.05). AAV-CNTF and crush injury (CNTF + 6wpc) resulted in RGCs that have fewer primary dendrites and less complex dendritic arbors than injury alone (Fig. 2*K–*M, #*p* < 0.05 6wpc vs CNTF + 6wpc; statistically significant difference detected at 30- to 90-µm radii but no other radii). Thus, these results indicate that acute optic nerve injury leads to reduction in αRGCs’ dendritic complexity, and CNTF plus injury causes an even further reduction of dendritic complexity in these neurons.

### Immunohistochemical staining of unsectioned whole optic nerve using iDISCO

**Figure 3.**
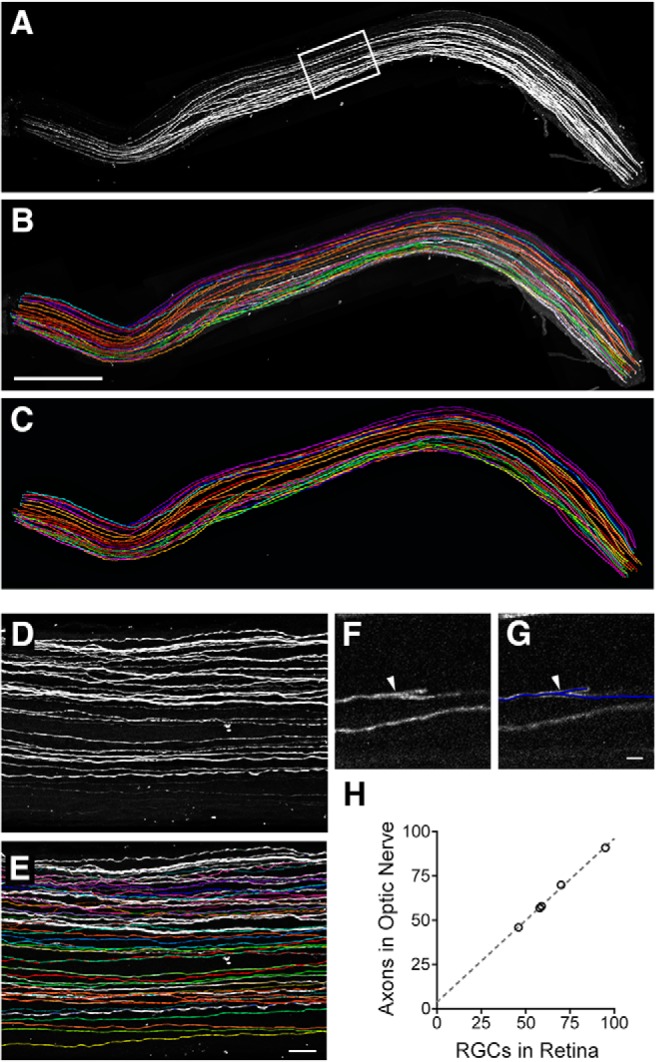
The iDISCO technique was used to immunostain unsectioned optic nerves from Thy1-H-YFP mice. ***A***, Maximum intensity projection (MIP) image of a full thickness optic nerve showing YFP^+^ axons (white). Orientation: optic nerve head on left, distal toward the optic chiasm to the right. ***B***, Traces superimposed on MIP image. Scale bar, 500 µm. ***C***, Example traces of single YFP^+^ axons, each color represents one continuous axon. Color assignment was arbitrary. ***D***, ***E***, High-magnification view of boxed area in ***A***. Scale bar, 30 µm. ***F***, Example of an uninjured axon branch. ***G***, Trace overlay of ***F***. Scale bar, 10 µm; arrowhead, branch point. ***H***, Scatter plot of the number of RGCs counted in the retina (abscissa) versus the number of axons counted in each optic nerve (ordinate) per animal, *n* = 5; dashed line, linear fit.

**Figure 4.**
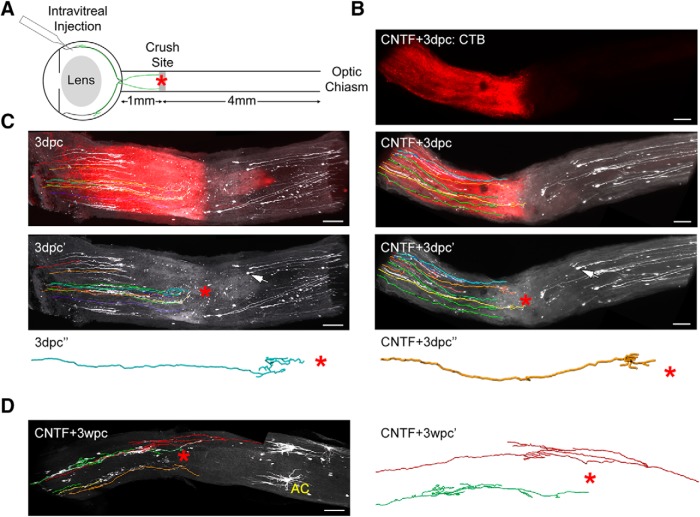
iDISCO based 3D analysis of Thy1H-YFP axons following optic nerve crush. ***A***, Diagram of the eye and optic nerve, indicating the location of intravitreal injection and the site of optic nerve crush. Two RGCs shown in green. Approximate distance of crush site to optic disk, and to optic chiasm are shown. Observed distance *in vivo* may vary ± 0.2 mm. ***B–D***, Maximum intensity projection (MIP) images of full thickness optic nerves showing YFP^+^ axons (white). Traces for YFP^+^ axons are shown with MIP image. Orientation, optic nerve head on left, distal toward the optic chiasm to the right. ***B***, Intravitreal AAV-CNTF-injected optic nerve 3dpc (CNTF + 3dpc), *n* = 4. ***C***, Optic nerve 3dpc (i.e., no AAV-CNTF), *n* = 4. ***B***, ***C***, One hour postcrush mice received intravitreal injection of CTB-Alexa Fluor 594 (red). Arrows in ***B***, ***C*** indicate a disconnected stump of distal degenerating axon. ***D***, Intravitreal AAV-CNTF-injected optic nerve 3wpc (CNTF + 3wpc), *n* = 4. AC (yellow) marks an example of a presumed astrocyte. ***B–D***, Lesion site indicated by red *. Each color represents an individual axon. Color assignment was arbitrary. Scale bar, 100 µm. Single axon traces are presented for select axons that displayed growth.

**Figure 5.**
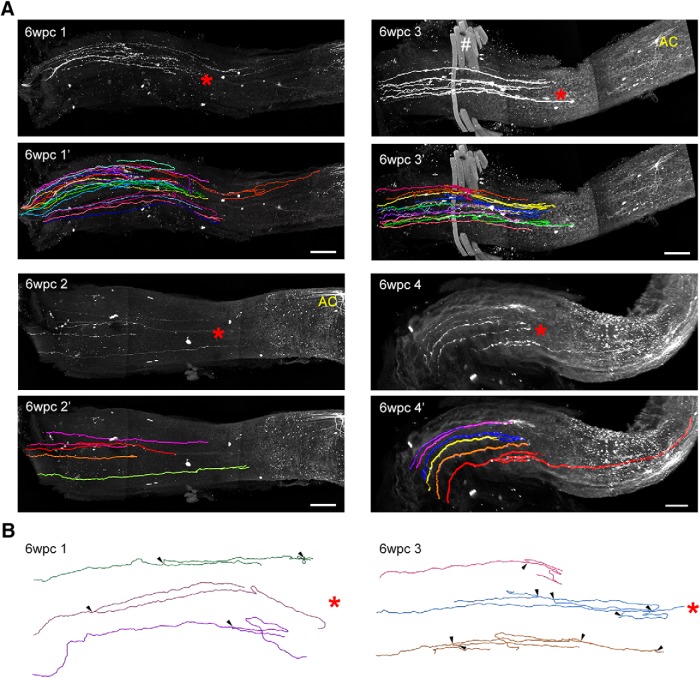
iDISCO based 3D analysis of YFP^+^ axons six weeks following optic nerve crush (6wpc). ***A***, Maximum intensity projection (MIP) images of full thickness optic nerves showing YFP^+^ axons (white). Lesion site indicated by red *. Orientation, optic nerve head on left, distal toward the optic chiasm to the right. Four optic nerves were members of this group, 6wpc 1–4. Traces for YFP^+^ axons are shown with MIP image. Each color represents an individual axon. Color assignment was arbitrary. Scale bar, 100 µm. AC (yellow) in 6wpc 2 and 6wpc 3 indicate examples of presumed astrocytes. White # in 6wpc 3: extraocular muscle that was not completely removed during dissection. ***B***, Three axons from 6wpc 1 and 6wpc 3 are shown. Each axon grows but fails to cross lesion site. Branch points marked by arrowheads. Lesion site indicated by red *.

To visualize and follow single axons throughout the optic nerve, we subjected adult Thy-H-YFP mouse optic nerves to a tissue clearing procedure which renders tissues transparent and facilitates whole tissue 3D imaging. Since tissue clearing procedures generally reduce endogenous YFP signal, we also immunostained the whole nerves using an antibody against YFP before tissue clearing using the iDISCO technique Representative images of uninjured optic nerve subjected to iDISCO and whole tissue imaging are shown in Figure 3*A–E*. Sparsely labeled individual YFP^+^ RGC axons are clearly visible. These axons project linearly from the optic disk to the distal optic nerve. In rare occasions, we also noticed that some axons have a short, rapidly terminating branch (Fig. 3*F*,*G*). We also observed occasional YFP^+^ cells and their processes in the optic nerve. These are likely astrocytes based on their morphology (Figs. 4*D*, 5*A*; AC, yellow). We have established the use of iDISCO to visualize the entire course of Thy1-H-YFP RGC axons through the optic nerve.

Next, we used iDISCO to evaluate how individual axons within a defined population of RGCs regenerate. To determine the validity of our method, we compared the number of RGCs to the number of axons in the optic nerve for five animals. Consistent with the number of YFP^+^ RGCs in the retina, there were ∼70 axons in each nerve. Furthermore, we find a strong correlation between the number of RGCs and axons (*R* ^2^ = 0.9975, *p* < 0.0001, *n* = 5 retina, optic nerve pairs; Fig. 3*H*), and on average we identified 98.5% of the axons predicted by the number of YFP^+^ RGCs. These data indicate that we can identify all YFP+ axons within the optic nerve.

### Analysis of single YFP^+^ RGC axons following optic nerve injury

To examine the morphology and growth pattern of YFP^+^ RGC axons following crush injury, first we collected Thy1-H-YFP mouse optic nerves 3dpc and performed iDISCO and 3D confocal imaging (Fig. 4*B*,*C*). To determine how an individual axon regenerates, we traced entire axons of some RGCs (Fig. 4*B*,*C*). Additionally, to visualize all RGC axons, CTB conjugated to Alexa Fluor 594 was injected 1 h after crush to label axons anterogradely. Even at this early stage (i.e., 3dpc; Fig. 4*C*, *n* = 4), some axons regrew within a small area near the cut end. However, there were no axons beyond the lesion site. Axons in the animals subjected to AAV-CNTF and crush injury (CNTF + 3dpc, *n* = 4) appear similar at this time point (Fig. 4*B*). Disconnected YFP^+^ axons which have not yet undergone Wallerian degeneration are visible distal to the lesion site (Fig. 4*B*,*C*, arrows indicate disconnected bulbs). All YFP^+^ axons are clearly disconnected, and no CTB labeled axons are found far distal to the lesion site (i.e., 1 mm distal to lesion site), strongly indicating that the injury is complete and no axons are spared from axotomy. These results show that some YFP^+^ axons begin growing soon after injury (3dpc). Notably, this initial growth appears independent to the presence of CNTF.

Second, we observed the growth pattern of YFP^+^ axons at an intermediate time point. At 3wpc, YFP^+^ axons in AAV-CNTF-treated animals (CNTF + 3wpc, *n* = 4 animals) regrew within regions proximal to the lesion site, forming complex branched and looped structures. Some axons also grew past the lesion site (Fig. 4*D*).

To further investigate this complex growth pattern, we examined axon regeneration at six weeks after injury. Optic nerves from four individual animals (6wpc 1–4) are shown in Figure 5. At six weeks postinjury (6wpc), nearly all surviving YFP^+^ RGC axons were limited to the region proximal to crush site, consistent with the limited ability of CNS axons to regenerate beyond the injury site. We note that the proximal edge of the lesion site occurs at ∼1 mm (±0.2 mm) away from the optic disk. In one animal, we observed one YPF^+^ axon past the lesion site, elongating to ∼1 mm away from the lesion site (Fig. 5*A*, 6wpc 4). However, we did not observe regenerating YFP^+^ RGC axons far beyond the lesion site (i.e., 3 mm from the lesion), indicating that the crush injury was complete and did not leave axons spared.

**Figure 6.**
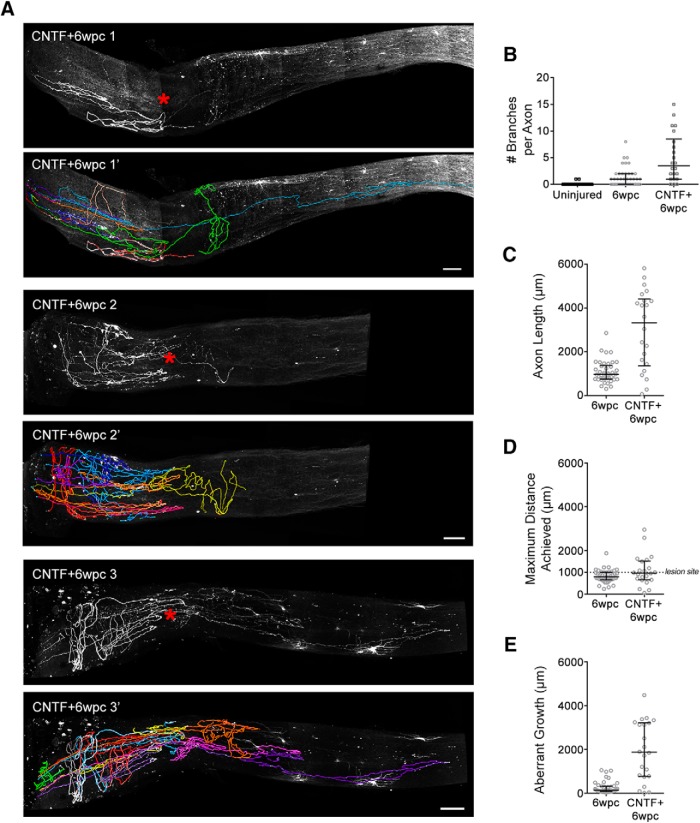
iDISCO based 3D analysis of YFP^+^ axons six weeks following AAV-CNTF injection and optic nerve crush (CNTF + 6wpc). ***A***, Maximum intensity projection (MIP) images of full thickness optic nerves showing YFP^+^ axons (white). Lesion site indicated by red *. Orientation, optic nerve head on left, distal toward the optic chiasm to the right. Four optic nerves were members of this group, CNTF + 6wpc 1–4. Only animals CNTF + 6wpc 1–3 had axons that could be fully traced and were included in the quantification. Traces for YFP^+^ axons are shown with MIP image. Each color represents an individual axon. Color assignment was arbitrary. Scale bars, 100 µm. ***B***, Dot plot of the number of branches occurring per axon in each group, each dot represents one axon. ***C***, Dot plot of axon total length for each axon. ***D***, Dot plot of the maximum distance each axon was found from the retina/optic nerve head boundary. Dashed horizontal reference line indicating lesion site at 1000 µm. ***E***, Dot plot of aberrant growth for each axon. Bars, median and interquartile range. Uninjured *n* = 34 axons from three animals, 6wpc *n* = 42 axons from four animals, CNTF + 6wpc *n* = 22 axons from three animals (CNTF + 6wpc 1–3).

**Figure 7.**
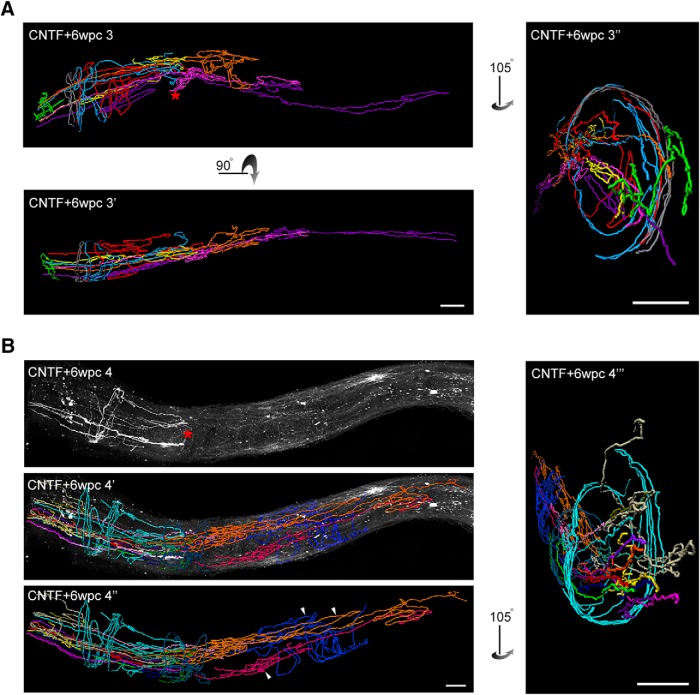
iDISCO based 3D visualization of aberrant axon growth. ***A***, Axon traces from animal CNTF + 6wpc 3 are shown from three perspectives. CNTF + 6wpc 3 and CNTF + 6wpc 3’ show longitudinal views of the same optic nerve. CNTF + 6wpc 3’’ and CNTF + 6wpc 4’’’ show coronal views of the optic nerve. ***B***, Animal CNTF + 6wpc 4. Continuous axon segments that could be traced with a high degree of certainty are shown. Each color represents a continuous segment. Lesion site indicated by red *. Arrowheads mark the three segments that grew beyond the lesion site. Scale bars, 100 µm.

Finally, we examined the response of YFP^+^ RGC axons six weeks following optic nerve crush when the animals received AAV-CNTF injection. Previous studies have demonstrated that CNTF allows some RGC axons to regenerate long distances in the optic nerve with some axons reaching the brain (Pernet et al., 2013; Yungher et al., 2015). We injected AAV-CNTF 3 d before unilateral crush, and we collected Thy1-H-YFP mouse optic nerves at six weeks after injury. Injured optic nerves from four individual animals are shown in Figures 6*A*, 7. Three of the four animals (CNTF + 6wpc 1–3) had axons that could be traced in their entirety. The fourth had extensive aberrant growth that prevented the tracing of each axon with certainty (Fig. 7*B*, CNTF + 6wpc 4); however, many axon segments could be traced effectively.

Tracing each axon in its entirety allows comprehensive axon pattern analysis. We observed that axon branching occurs frequently following crush. Approximately half of the axons have 1 or more branches (Fig. 6*B*, 6wpc). AAV-CNTF significantly increased the number of axons with branches (*p* < 0.05) and the number of branches per axon (*p* = 0.0001; Fig. 6*B*, 6wpc vs CNTF + 6wpc). Next, we sought to characterize for each axon (1) total axon length, (2) how far centrally an axon reached (maximum distance achieved from the optic disk), and (3) how much aberrant growth occurred (aberrant growth = axon total length – maximum distance achieved; Fig. 6*C–E*). Given that the proximal edge of the crush site is ∼1 mm from the optic disk, we see that most axons in the injury control animals remain within or near the lesion site (i.e., within 0.8–1.2 mm from the optic disk). Surprisingly, even in the absence of growth promoting factors many of these axons grew (Figs. 5*B*, 6*C* ). This growth was mostly within the region proximal to the crush. Some axons grew between 1 and 2 mm in length. This growth consists of branches and loops, growth that does not extend the axon distally along the optic nerve (Figs. 5*B*, 6*E*). Together, these results show that YFP^+^ RGCs are innately capable of regrowing long axons; however, these axons are unable to successfully traverse the lesion.

Consistent with the regeneration promoting effects of CNTF, axons grew significantly longer than controls (Fig. 6*C*, *p* < 0.001). Like the injured control animals, many axons failed to grow through the lesion site, and most regrowth was aberrant (Fig. 6*E*). In CNTF animals, the axon length measurement shows that despite growing >4–5 mm, some axons are restricted to the lesion area. Independent of treatment, the maximum distance along the optic nerve most growing axons achieve is associated with the lesion site, around 1000 µm from the optic disk (Fig. 6*D*, distance of lesion site from optic disk indicated as horizontal reference line at 1000 µm). In fact, the median maximum distance achieved by axons in CNTF animals was only 25% longer than that of the control injured animals despite the fact that the CNTF axons grew 3- to 4-fold longer in total length (Fig. 6*C–E*). Figure 7*A* shows reconstructed axons of the CNTF + 6wpc 3 optic nerve from different viewing angles. This pattern of regrowth was also evident in the fourth animal (Fig. 7*B*, CNTF + 6wpc 4). While we were unable to trace each axon from the optic nerve head to termination, we determined that no more than three axons grew beyond the lesion site (Fig. 7*B*, arrowheads). As shown in the cross view of the optic nerve (Fig. 7, images in the right panels), some axons wrap around the nerve several rounds, further illustrating the extensiveness of tortuous growth.

## Discussion

RGCs of different subtypes are connected to distinct presynaptic partners and exhibit an array of responses to visual stimuli. RGCs of different subtypes also project their axons to different brain targets and contribute to image-forming functions as well as non-imaging forming functions (Sanes and Masland, 2015) Anatomically, how do the dendrites and axons of specific RGC types respond to axotomy, as well as after treatments with factors that stimulate axon growth? In the retina, several studies have characterized RGC type-specific changes in dendrite morphology after traumatic axotomy or in glaucoma models. As such, changes in dendrites after insult have been described to some extent. However, the abilities of specific RGC types to regenerate axons and correctly find their targets in adult mammals are just beginning to be determined. Since some studies have shown that many regenerating RGC axons grow circuitously near the lesion and fail to regenerate far, we sought to combine sparse neuronal labeling with iDISCO and follow individual axons derived primarily from one RGC type; αRGC. The major observations in this study are (1) αRGC dendrites decrease their complexity following axotomy, and this response to axotomy is exacerbated by CNTF treatment; (2) YPF^+^ RGC axons grow over unexpectedly long distances before the lesion site with only a few axons being able to successfully traverse the lesion; and (3) YFP^+^ axons that do regenerate beyond the lesion site elongate aberrantly, form many collateral axons in the optic nerve and fail to reach the brain.

### Considerations for cell types of origin of the YFP^+^ processes

Our results show that the majority of surviving YFP^+^ RGCs after injury are immunoreactive for OSPN, indicating that most of these YFP^+^ RGCs belong to αRGCs (Duan et al., 2015; Sanes and Masland, 2015). Together with the observation that αRGCs have higher capacity to regenerate axons compared to other RGCs in general (Watanabe and Fukuda, 2002; Duan et al., 2015), we reason that the majority of YFP^+^ RGC axons that we analyzed are likely to be of αRGCs. However, because 24% of surviving RGCs are OSPN-negative (i.e., of the 13 surviving YFP^+^ RGCs per retina, 10 are YFP^+^/OSPN^+^ and 3 are YFP^+^/OSPN^−^), we are unable to conclusively determine if the axons that grew beyond the lesion site are of αRGC origin.

### Changes in αRGC dendrites in responses to axotomy and CNTF

Using transgenic mice that label defined RGC types, previous studies have examined the morphologic changes that occur in RGC dendrites. In a mouse glaucoma model, OFF transient RGCs showed decreased dendritic arborization (Della Santina et al., 2013). Similarly, the dendritic complexity of transient OFF αRGCs from glaucomatous mouse eyes is reduced (El-Danaf and Huberman, 2015). Thus, the results in our study showing loss of dendritic complexity in αRGCs are in line with these previous reports. In contrast, in adult rats, peripheral nerve graft and AAV-CNTF cause an increase in soma size of RGCs without affecting the dendritic complexity or field size (Rodger et al., 2012). However, when examined specifically in the RGC 1 subtype (i.e., RGCs with a large soma), AAV-CNTF caused a significant reduction in the complexity of the dendritic arbors, again without reducing the field size (Rodger et al., 2012). In line with the previous study, our results show that AAV-CNTF leads to a significant decrease in arbor complexity without altering the dendritic field size in most cells. Thus, our results suggest that while αRGC axons are highly regenerative and quite likely generate complex arbors (i.e., in response to CNTF), their dendrites become less complex. In this regard, one could ask why and how does CNTF cause even greater reduction in dendrite complexity in these RGCs? The molecular and cellular mechanisms underlying such different behaviors by the axons and dendrites are unknown. It is also unclear whether the reduction in dendrite arbor complexity will be potentially disruptive for the function of these RGCs (i.e., receive less input from their presynaptic partners). These are interesting questions that may deserve further investigation.

We observed that the majority of YFP-labeled αRGCs (∼80%) in this experimental example died at six weeks after injury. Currently, it is unclear whether these YFP^+^ αRGCs die long after injury because of lack of intrinsic survival signals or because they are disconnected for a long time and lack trophic support from the target. It is also possible that they die because they remain unmyelinated (i.e., not remyelinated) for a long period time, lacking the survival signal(s) and other type(s) of support from the oligodendrocytes (or even astrocytes). The question why most RGCs die remains nebulous and justifies further investigation.

### Highly regenerative yet unable to go far

Several prior studies have adopted tissue clearing strategies and examined axon regeneration in unsectioned, whole CNS tissues (Ertürk et al., 2011; Laskowski and Bradke, 2013; Luo et al., 2013; Pernet et al., 2013; Soderblom et al., 2015). In this study, we sought to expand the 3D analysis to track single axons in given neuronal types. To our knowledge, our study is the first to report the adaptation of sparse labeling and whole tissue staining to attain the entire projection profiles of regenerating axons. Perhaps the most striking observation in our study was the extensive and circuitous regeneration of RGC axons occurring proximal to the lesion site. Axons start to penetrate through the lesion, sometime more than once, but each time they turn back toward the retina and thus they elongate within the proximal optic nerve region (i.e., near the optic nerve head). Additionally, axons that surpass the lesion continue to branch and misroute, with these events lacking an obvious spatial pattern (e.g., occurring near the lesion site). We also note that none of these axons grew past the optic chiasm. As can be seen in Figures 4–7, regenerating axons stop at different distances away from the chiasm. Previous studies have suggested that optic chiasm may inhibit or halt some axons to grow further (Luo et al., 2013; Crair and Mason, 2016). At least for these neurons however, the reason that they fail to grow past the chiasm does not seem to be due to chiasmatic barrier as these axons terminate or turn toward the eye even before they get close to the optic chiasm.

How does an axon successfully traverse the lesion? In the CNS lesion, various growth inhibitory molecules are present. Molecular barriers within the lesion area include chondroitin sulfate proteoglycans and myelin-associated inhibitors (Yiu and He, 2006). Astrocytes and fibroblasts interact to establish a scar, surrounding the lesion. Therefore, to successfully traverse the lesion, a growing axon will need to modify the extracellular matrix (ECM) and overcome inhibitory/repulsive cues. Differential expression of ECM modifying enzymes and cell surface receptors may explain why some axons never cross the lesion while others do. This simple explanation is challenged by our finding that axons proximal to the lesion form extensive branches and loops and then eventually grow through the lesion (i.e., Fig. 6*A*, CNTF + 6wpc 1). For this to occur, axons would need to be dynamically responsive to their environment and alter gene expression accordingly (i.e., after failing to traverse the lesion, they change expression of certain molecules to better suit the lesion environment), or crossing the lesion site is a stochastic event. Future investigation will be needed to identify if specific RGCs are can cross the lesion barrier, and what interventions can help axons through this environment.

The tracing of individual axons allowed us to measure the total length of individual axons as well as the maximum distances from the eye. Unequivocally, we find that while many axons travel long distances, their circuitous growth results in them remaining within close vicinity to the lesion site. The observation that the regenerating axons grow aberrantly within the optic nerve is mostly in agreement with previous studies (Luo et al., 2013; Pernet et al., 2013). Nonetheless, our current study provides a complete picture of individual axon trajectory, from their entry into the optic nerve to the end of their route. This is particularly the case for the portion of axons located in the proximal area to the injury site which was not possible in the previous studies that have used CTB as a tracer for all RGC axons. Overall, our results build on prior studies and further show that the growth of YFP^+^ RGCs axons are tortuous in their paths, and that these axons are unlikely to reinnervate their brain targets after CNTF treatment.

### Question of treatment and cell type-specific axon behaviors

CNTF is only one of many factors that can induce axon regeneration. Do different regeneration stimulating factors cause a similar degree of aberrant growth and misrouting? Do the axons of all RGC types have similar propensity to misroute? Studies by Lim et al. (2016), and de Lima et al. (2012), showed that at least under certain conditions, some RGC axons are able to regenerate with minimal misrouting and back to their correct targets. In some instances, these RGC axons seem to travel linearly toward the brain (Lim et al., 2016). It appears that under some conditions, RGC axons may be able to navigate through the injured optic nerve. It is unknown what molecular and cellular factors minimize misrouting and produce directed growth within the optic nerve and beyond. It may be interesting to apply 3D analysis and examine the behavior of αRGCs and other RGC types under additional growth-promoting conditions. This could help determine if the aberrant growth seen in the present study is specific to some RGC types or to CNTF treatment per se.

It is known that certain types of neurons in different CNS regions including the supraspinal serotonergic neurons are able to spontaneously remodel and reextend axons after axotomy (Hawthorne et al., 2011). On the other hand, other neurons including the corticospinal tract axons are strongly refractory to regeneration where the cut dystrophic axons regress with virtually no signs of regrowth (Thallmair et al., 1998; Liu et al., 2010). In the case of RGCs, we know from studies using CTB tracing that a few of these neurons can spontaneously regrow axons, at least to some short distance into the lesion. The lengthy axons seen even without CNTF in our study indicate that RGCs (and perhaps other CNS neurons) may have much higher growth capacity than generally thought. In the case of peripheral neurons, the dorsal root ganglion (DRG) axons send collaterals and misroute following injury to the central branch (Kerschensteiner et al., 2005). It would be interesting to determine if this aberrant growth of DRG axons is amplified following preconditioning injury, a method to promote regeneration of a DRG’s central branch.

These findings highlight the need for prudence when evaluating a growth factor to promote axon regeneration. CNTF and other growth factors are frequently used in regeneration studies to stimulate axon growth. In our study, CNTF produced abundant axon growth, but this growth was highly aberrant. Thus, this growth factor caused “too much of a good thing.” Therefore, future treatments must be adjusted to maximize axon growth into brain targets, and minimize axon branching and tortuous growth.

Overall, our study documents the morphologic changes that occur in αRGCs after optic nerve injury. Tracking the entire paths of individual axons reveal that these RGCs can naturally re-extend axons extremely well, but both their inability to traverse the lesion area and their circuitous axon growth limit reconnection with the brain. Our results counter the general view that RGC axons are incapable of lengthy regeneration, and shift the focus from promoting axon elongation, to understanding factors that prevent direct growth of axons through the injured nerve.

## Synthesis

Reviewing Editor: Esther Stoeckli, University of Zurich Decisions are customarily a result of the Reviewing Editor and the peer reviewers coming together and discussing their recommendations until a consensus is reached. When revisions are invited, a fact-based synthesis statement explaining their decision and outlining what is needed to prepare a revision will be listed below. The following reviewer(s) agreed to reveal their identity: Eloisa Herrera, Carol Mason.

We have discussed the evaluations of your manuscript and come to the conclusion that the study technically is very well done and has its potential. This manuscript is a welcome study on retinal ganglion cell (RGC) axon regeneration after optic nerve crush. You put a long-known Thy1-H-YFP line to good use: RGCs, their axons, and dendrites are labeled but only in a sparse population, the majority of which are osteopontin-expressing alpha RGCs. The path of these axons is traced several weeks post crush to demonstrate that axons can regrow proximal to and beyond the crush, but not without circling and doubling back in many cases. The study demonstrates that the application of CTNF, a growth factor known to stimulate axon regrowth, made such misrouting even more severe. Dendrites in both crush situations were diminished in several parameters, even more after CTNF treatment. The data are very clearly presented, aided by tissue clearing with iDisco and by computer-assisted axon tracing.

However, we also felt that the potential of the study has not been exploited. In essence it has been shown before that axons of retinal ganglion cells (RGCs) can regenerate over long distances. Also the finding that 76% of the regenerating fibers are of the OSPN type is not surprising and does not indicate enhanced regeneration capacity given the fact that they make up 70% of the RGCs. Therefore, these aspects are not novel and do not go beyond what is already known. Having that said we think that your results do contribute to our understanding of RGC regeneration but these aspects have not been described very well. For instance, your approach could be used to analyze whether the crush lesion does spare any axons, or in other words, how uniform the crush lesion is. The Thy-1 transgenic mouse used in this study provides a valuable model to follow up on axonal behavior in a way not possible before, because individual axons can be traced over a very long distance. Therefore, we recommend that you describe the details of the lesion much better and use it to follow up on axonal behavior at different time points after lesion. It is interesting to see that the behavior of axons and dendrites differs markedly. Again, this issue has not been described very well. In fact, at first it even seems confusing why the results of Figure 2 and Figure 5 show opposite outcomes. The manuscript would benefit from rewriting and the inclusion of clear statements at the end of paragraphs. Given that the manuscript uses a novel technique to repeat some previously published studies, it would be helpful to clearly comment on where the present study confirms and where it contradicts those studies.

Please pay attention to the following issues raised by the reviewers:

Reviewer 1:

- The number of animals they use per experiment use to be four. I believe they should increase the “n” in order to reach conclusions.

- Drawings indicating how do they practice the crash and arrows or lines indicating where the crash was practiced would help the reader to better follow the story.

- I am not sure why do they insist so much in the fact that the RGCs most resistant to the crash are the alphaRGCs. First, this has been already published (as themselves point out) and second, it is expectable because the majority of the RGC labeled in the transgenic mice are precisely the alphaRGCs.

Reviewer 2:

There are a number of textual and other amendments to the figures that would improve the presentation:

1.Methods - indicate the post-crush survival times that were evaluated.

2.Figures:

a. The crush site should be designated in all figures; it is difficult to discern where the crush is in most micrographs.

b. Lines 192-194: The authors describe that AAV-CNTF delivery combined with crush injury resulted in RGCs that have fewer primary dendrites and less complex dendritic arbors than injury alone (Figure 2K-M; “Crush vs CNTF + Crush”, p < 0.05). In Figure 2M, authors should clarify their measurement method and results stating that they were “significantly different between Crush and CNTF + Crush”. The difference between Crush and CNTF + Crush can be detected only at the 60-90um radius, but not at other radii. An asterisk should be placed to indicate at which radii there is a statistically significant difference.

c. The optic chiasm is the barricade to growth in most published studies on RGC regeneration after optic nerve crush. Do all axons stop at the same point distal to the crush site? If so, how close/far is this site from the start of the chiasm?

d. Line 540 - there is no dashed line indicating the lesion site.

3.Optic nerve crush may is not an ideal method to damage axons, because every crush may not be uniform, but this method remains the major model for optic nerve regeneration.

a. The authors have an opportunity with this YFP mouse to assess whether their technique is efficient, i.e., that crush/axon severing is complete. Have the authors viewed the nerve shortly after crush (hours, a few days)? Most of the data shows 6 weeks post-crush. Comment and illustration of this issue would be helpful.

b. To supplement an earlier time course analysis, antibodies to GAP-43 could be used to verify that axons are growing and not just resistant to injury. This may require additional work, but the authors should comment on this suggestion, as it relates to a. above.

4. Duan et al., (2015) demonstrated that ~80% and then ~50% of alpha RGCs can survive 14 days (2 weeks) or 28 days (4 weeks), respectively, after optic nerve injury. In the present paper the authors show that 20% of alpha RGCs (10/49 YFP+/ OSPN+ RGCs) survive 6 weeks after injury. Together, these data indicate that even alpha RGCs die over time post-injury. The authors might discuss whether intrinsic survival signals decrease and/or extrinsic influences such as inflammation, or the inability to connect to their targets, affect survival of alpha RGCs in the long term.

## Author Response

We thank the Editorial Board Member and the two Reviewers for their words of appreciation and for their insightful and constructive comments. We accepted all criticism and have revised the manuscript accordingly. The changes we made are emphasized in red font in the manuscript and detailed point-by-point below.

Editorial Board Comments:

- Having that said we think that your results do contribute to our understanding of RGC regeneration but these aspects have not been described very well. For instance, your approach could be used to analyze whether the crush lesion does spare any axons, or in other words, how uniform the crush lesion is. The Thy-1 transgenic mouse used in this study provides a valuable model to follow up on axonal behavior in a way not possible before, because individual axons can be traced over a very long distance. Therefore, we recommend that you describe the details of the lesion much better and use it to follow up on axonal behavior at different time points after lesion.

In our revised version, we provide a more easily understood description of the lesion with a diagram in Figure 4. We also generated more animals by examining the axon behavior at 3 days and 3 weeks after crush injury, in addition to the 6 weeks post crush in the original version.

It is interesting to see that the behavior of axons and dendrites differs markedly. Again, this issue has not been described very well. In fact, at first it even seems confusing why the results of Figure 2 and Figure 5 show opposite outcomes. The manuscript would benefit from rewriting and the inclusion of clear statements at the end of paragraphs.

We added more detailed descriptions regarding the behavior of dendrites (in comparison to that of the axons) and its potential implication (text lines 200 -202 and 331-338), and provided more explicit statements at the end of each major paragraph (e.g. lines 185-187, 200-202, 214-215, and 222)

Given that the manuscript uses a novel technique to repeat some previously published studies, it would be helpful to clearly comment on where the present study confirms and where it contradicts those studies. Please pay attention to the following issues raised by the reviewers:

In this study, we reveal the precise growth pattern of individual axons proximal and distal to the lesion site. The overall conclusion is that while regenerating axons grow aberrantly within the optic nerve distal to the injury (in agreement with previous studies cited within the manuscript), there is an unexpectedly abundant and tortuous growth of regeneration axons proximal to the injury. It was not possible to observe this previously in studies that used CTB as a tracer for all RGC axons. Remarkably, animals that did not receive regenerating factors exhibit regenerative axon growth as early as 3 days, the earliest point we examined, and they do not remain static. Furthermore, axons that escape the lesion site have strikingly abnormal routing that was not as appreciated in the previous studies. We included these points in the Discussion section (lines 381-386) to emphasize the benefit of the improved technique used in this study and how it helped us expand on the previous studies.

Reviewer 1:

- The number of animals they use per experiment use to be four. I believe they should increase the “n” in order to reach conclusions.

As the reviewer has correctly pointed out, we analyzed 4-5 animals in each of the injured groups (i.e. Crush + CNTF and Crush, 6 weeks after crush injury). The two main conclusions we have reached from these animals are that; i) the RGCs in both groups re-extend damaged axons quite extensively prior to the injury site, and at the distal region to the injury site, the axons in the Crush + CNTF group travel long distances without direction, and ii) the sparse cell labeling and whole tissue immunohistochemistry/imaging methods that we have adopted in this study are a technical expansion to previous methods and permit precise phenotypic analysis of individual regenerating axons. While it could be useful to add more animals in these groups, we think that the number of animals used is sufficient for us to reach these main conclusions. We feel confident in these conclusions due to the magnitude of the effect we observed. Having said that, as suggested by Reviewer 2, we did generate more animals to examine axon behaviors at 3 weeks after injury, and at this time point, we also see very similar axon behaviors compared to the 6 weeks post injury animals, further validating the main conclusions. In the 8 animals (n=4 CNTF+3wpc, n=4 CNTF+6wpc) that we have assessed, we have consistently observed similar axon growth behaviors. We also analyzed the behavior of animals 3 days after crush showing that the growth of axons is in both control and CNTF treated is an early and intrinsic effect. We have included results from these additional animals in the revised manuscript (Figure 4).

- Drawings indicating how do they practice the crash and arrows or lines indicating where the crash was practiced would help the reader to better follow the story.

We have now added a diagram to visualize better the injury model used in this manuscript (Figure 4 in the revised manuscript).

- I am not sure why do they insist so much in the fact that the RGCs most resistant to the crash are the alphaRGCs. First, this has been already published (as themselves point out) and second, it is expectable because the majority of the RGC labeled in the transgenic mice are precisely the alphaRGCs.

As the reviewer has pointed out, previous studies have reported that alpha RGCs tend to survive and regenerate better than other RGC types after axotomy. We would like to emphasize that it was not our intension to highlight these unique properties of alpha RGCs as new findings. Rather, we sought to underscore that this Thy1 mouse line does label this RGC type (which make it possible to assess axon regeneration in few RGCs) and more importantly, establish the feasibility of examining how axons from a specific RGC type would behave after injury and CNTF. Nonetheless, we agree with the reviewer about this point, and accordingly we removed or toned down the statements and description that emphasizes the unique injury responses of alpha RGCs (text lines 172 and 342 in the original version).

Reviewer 2:

There are a number of textual and other amendments to the figures that would improve the presentation:

1.Methods - indicate the post-crush survival times that were evaluated.

We indicated the post-crush survival times in the Methods and Results sections. Since we added more time points in this revision (i.e. 3 days and 3 weeks post-crush survival times), we now describe animal groups in these time points more explicitly to avoid confusion. 3 days post-crush, 3dpc; 3 weeks post-crush, 3wpc; 6 weeks post-crush, 6wpc.

2.Figures:

a. The crush site should be designated in all figures; it is difficult to discern where the crush is in most micrographs.

We included an asterisk to indicate the crush site in all figures.

b. Lines 192-194: The authors describe that AAV-CNTF delivery combined with crush injury resulted in RGCs that have fewer primary dendrites and less complex dendritic arbors than injury alone (Figure 2K-M; “Crush vs CNTF + Crush”, p > 0.05). In Figure 2M, authors should clarify their measurement method and results stating that they were “significantly different between Crush and CNTF + Crush”. The difference between Crush and CNTF + Crush can be detected only at the 60-90um radius, but not at other radii. An asterisk should be placed to indicate at which radii there is a statistically significant difference.

1. It was noted on our review of this figure that the error bars were incorrectly plotted at the 95% CI opposed to the indicated {plus minus}SEM. This has been corrected in figure 2M.

2. We initially used a 2-way repeated measures ANOVA to calculate group significance across all distances. We have now included 1-way ANOVA calculations at each distance, and have included significance indicators in 2M. p-values for each comparison have been added to the statistics table.

3. We added statement to clarify the measurement methods (lines 124-125) and included more explicit description stating the differences between the “Crush” and “CNTF + Crush” (now described as “6wpc” vs “CNTF + 6wpc”) (lines 197-200).

c. The optic chiasm is the barricade to growth in most published studies on RGC regeneration after optic nerve crush. Do all axons stop at the same point distal to the crush site? If so, how close/far is this site from the start of the chiasm?

In all animals (4 CNTF+3wpc mice, 4 6wpc, and 4 CNTF+6wpc) that we have analyzed, we did not observe axons growing past the optic chiasm. As can be seen in Figures 4-7, regenerating axons stop at different distances (e.g. ranging from approximately 0.5 to 4-5 mm) away from the chiasm. However, at least for these neurons, the reason that they fail to grow past the chiasm does not seem to be the chiasmatic barrier as the axons terminate or turn towards the eye even before they get close to the optic chiasm. In our revised version, we included a brief description about this aspect (lines 357-363 in the new version).

d. Line 540 - there is no dashed line indicating the lesion site.

Reference to the dashed line has been clarified in the figure legend: “Dashed line” has been changed to “Dashed horizontal reference line”. Additionally, a label was added to the reference line in figure 6D (revised version). We added a sentence to clarify this in text lines 280-283.

3. Optic nerve crush may is not an ideal method to damage axons, because every crush may not be uniform, but this method remains the major model for optic nerve regeneration.

a. The authors have an opportunity with this YFP mouse to assess whether their technique is efficient, i.e., that crush/axon severing is complete. Have the authors viewed the nerve shortly after crush (hours, a few days)? Most of the data shows 6 weeks post-crush. Comment and illustration of this issue would be helpful.

Based on the reviewer's suggestions, we generated additional animals to examine axon behavior at 3 days and 3 weeks after crush injury. In this revised version, we now include the results from these animals. See text lines 224-242, and Figure 4.

b. To supplement an earlier time course analysis, antibodies to GAP-43 could be used to verify that axons are growing and not just resistant to injury. This may require additional work, but the authors should comment on this suggestion, as it relates to a. above.

We tested GAP43 antibody to stain regenerating axons in this model. GAP43 antibody was a gift from Dr. Dietmar Fischer. Please see figure at the end of this document. Although there seems to be some background signal using this antibody, we observed regenerating Thy1H-YFP axons 2 weeks after optic nerve crush and AAV-CNTF injection. We observed Thy1-H+ CTB+ axon segments that show distinct Gap43 immunoreactivity, arrow heads. We also think it is hard to imagine how axons that are “just resistant to injury” could acquire these complex morphologies without also growing.

4. Duan et al., (2015) demonstrated that ~80% and then ~50% of alpha RGCs can survive 14 days (2 weeks) or 28 days (4 weeks), respectively, after optic nerve injury. In the present paper the authors show that 20% of alpha RGCs (10/49 YFP+/ OSPN+ RGCs) survive 6 weeks after injury. Together, these data indicate that even alpha RGCs die over time post-injury. The authors might discuss whether intrinsic survival signals decrease and/or extrinsic influences such as inflammation, or the inability to connect to their targets, affect survival of alpha RGCs in the long term.

The reviewer in this sentence raises several interesting points. As the reviewer has pointed out, the majority of alphaRGCs in this experimental example died at 6 weeks after injury. Currently, it is unclear whether these RGCs die because of lack of intrinsic survival signals or due to the fact that they are disconnected long term and lack trophic support from the target. It is also possible that they die because they remain unmyelinated (i.e. not remyelinated) for a long period time, lacking the survival signal(s) and other type(s) of support from the oligodendrocytes or even astrocytes). The question why most RGCs die remains nebulous and justifies further investigation. We have included these ideas in the discussion. See text lines 339-345.

Thy1H-YFP axons show Gap43 immunoreactivity. Optic nerve sections of animals treated with AAV-CNTF 3 days prior to crush. Cholera-toxin beta conjugated to Alexa-555 was inject 3 days prior to perfusion. Two weeks following optic nerve crush Thy1H-YFP axons and CTB+ axons are seen growing beyond the lesion site. Distinct GAP43 reactivity was observed in Thy1H-YFP+ CTB+ axons, arrowheads. Scale bars: 100µm, and 10µm for region of interest.
